# Influence of Citric Acid on the Fundamental Properties of CO_2_ Cured Magnesium Oxysulfate Paste

**DOI:** 10.3390/ma16031315

**Published:** 2023-02-03

**Authors:** Houchao Sun, Feiting Shi, Hui Wang

**Affiliations:** 1 School of Civil Engineering, Yancheng Institute of Technology, Yancheng 224051, China; 2 School of Civil and Environmental Engineering, Ningbo University, Ningbo 315000, China

**Keywords:** magnesium oxysulfate, CO_2_ curing, citric acid, mechanical strengths, erosion effect

## Abstract

Magnesium oxysulfate (MOS), mainly composed of magnesium oxide and magnesium sulfate, is a kind of gas-hardening cementing material with low energy consumption and CO_2_ emissions. In order to develop environment-friendly cement-based materials, MOS needs to be studied systematically. The paper mainly investigates the influence of citric acid (a retarder) on the working and mechanical properties of MOS paste. In this study, the setting time of fresh MOS paste is determined. The flexural and compressive strengths of hardened specimens exposed to the environment of water dry-wet (D-W) alternations, freeze-thaw (F-T) cycles, and sulfate D-W alternations are investigated. Furthermore, the drying shrinkage (D-S) rate of MOS paste is tested for 3 days and 28 days. The specimens are cured in standard or CO_2_ curing environments. A scanning electron microscope energy spectrum (SEM-EDS) is obtained to analyze the morphology of hydration products. Results show that citric acid can increase the setting time of MOS paste. The citric acid and CO_2_ curing have a positive effect on the mechanical strengths and the resistance to erosion by water, F-T cycles, and sulfate D-W alternations. The D-S rate decreased in relation to the increasing dosages of citric acid and increased with CO_2_ curing. MOS with 0.3% of the total binder material mass shows the best erosion resistance. As observed in the results of SEM-EDS, the CO_2_ curing and the citric acid can make the hydration products denser.

## 1. Introduction

Ordinary Portland cement (OPC) is one of the most widely used materials in the construction of houses, roads, and bridges [1]. However, the production process of OPC consumes a large amount of natural materials and energy, as well as releasing a large amount of carbon dioxide (CO_2_) [2]. Hence, developing and researching new cementitious materials with low energy consumption and low resource waste is necessary [3].

Magnesium oxysulfate (MOS) materials are prepared by mixing MgSO_4_ solution with light-burning active MgO [4]. The main mixing agent of MOS materials (magnesium sulfate) does not contain chloride ions [5], and hence the moisture absorption of traditional magnesium oxychloride-based materials will not occur [6]. As pointed out in Jia’s studies [7], the chloride-binding capacity of magnesium sulfate is higher than 74%. Therefore, the inner reinforcement of MOS is more resistant to chloride corrosion. Compared with magnesium phosphate-based materials, MOS materials have a longer final setting time [8]. As a result, excessive early hydration heat induced by rapid hardening cannot happen. Meanwhile, no toxic gas (CO_2_, NO_2,_ and SO_2_) is generated during condensation [9]. Wu et al. [10] have improved the volume stability by adding citric acid. Moreover, the citric acid can react with MgO to form a protective layer of insoluble magnesium citrate on the surface of MOS, leading to a delayed setting time [11]. Wang’s journal [12] pointed out that the compressive strength of MOS paste can be improved by adding citric acid. As concluded in Jin’s research [13], granite powder can effectively increase the water resistance coefficient of MOS paste. As reported by some studies, magnesium sulfate shows better thermostability compared to OPC [14]. In some studies [15], when the temperature is lower than 300 °C, the residual mechanical strength is higher than 60%. However, when hardened MOS materials are immersed in water, the mechanical strengths are clearly reduced [16]. The mechanical strengths of MOS decrease by 13.2%-50.93% after immersion in water for 28 days [17]. Therefore, improving the water resistance of MOS is an important technical problem [18]. Although the water resistance of MOS paste has been investigated by several researchers, little attention has been paid to its resistance to freeze-thaw cycles.

The main problem that plagues human lives in the world today is global warming [19], and CO_2_ is one of the main gases responsible for the greenhouse effect. Several efforts have been reported to reduce the emissions of CO_2_ [20]. As proved in the studies of Li et al. [21], cementitious materials can be used to reduce the concentration of emitted CO_2_. At the same time, CO_2_ curing on cement matrix improves the mechanical strength and durability of cement-based materials because CO_2_ can react with Mg(OH)_2_ [22] or Ca(OH)_2_ [23] to form MgCO_3_ or CaCO_3_ products, which improve the compactness of hydration products. Furthermore, the CO_2_-cured reinforced concrete has been found to decrease the corrosion area rate to 47.3% of the standard-cured reinforced concrete, leading to improved corrosion resistance [24]. The CO_2_ curing method not only improves the properties of materials but also consumes CO_2_ [25].

MOS can react with CO_2_ to form magnesium carbonate, which eventually leads to higher mechanical strengths and durability. Consequently, CO_2_ curing with citric acid is required to test the mechanical strengths and durability of MOS-based materials. However, little research about this is reported. The innovation of this research is the influence of citric acid on the working, mechanical properties and freeze-thaw resistance of CO_2_-cured magnesium oxysulfate paste.

In this study, the setting time of MOS with citric acid ranging from 0% to 0.4% by ratio of the total mass of MgSO_4_ and MgO is determined. The flexural and compressive strengths of MOS mortar cured in a standard or CO_2_ curing environment for 3 days and 28 days are measured. The drying shrinkage (D-S) rate is determined. The influence of water dry-wet (D-W) alternations and water freeze-thaw (F-T) cycles on the mechanical strengths of MOS mortar are considered. This paper will provide a new energy-saving and environmentally friendly cement-based material that will be used in civil engineering construction.

## 2. Experimental

### 2.1. Raw Materials

Citric acid (C_6_H_8_O_7_), magnesium sulphate (MgSO_4_·7H_2_O), and light magnesium oxide (MgO) provided by Yesheng Environmental Protection Technology Company, Louyang, China; Henan Huakai Biotechnology Co., Ltd., Luoyang, China; and Hebei Gaochang Chemical General Factory, Gaochang, China, respectively, are applied in manufacturing the MOS. The purities of citric acid, magnesium sulphate, and light magnesium oxide are 99.6%, 99.3%, and 99.1%, respectively.

### 2.2. Sample Preparation

The following experimental steps are used for preparing the specimens: The NJ-160 cement mixer is used for stirring the magnesium sulphate and light magnesium oxide. First, stir for 30 s at 140 r/min. Next, water is added to the cement mixer and mixed at 140 r/min for 90 s. After that, the fresh paste is mixed at 285 r/min for another 120 s. Finally, the fresh paste is used for measuring the setting time and the slump flow. The residual fresh paste is poured into the molds, forming specimens with dimensions of 40 mm × 40 mm × 160 mm. The mixing proportions of the specimens are shown in Table 1. In order to provide a comparison with previous studies, the amount of citric acid ranges from 0% to 0.4% by the ratio of the total masses of MgO and MgSO_4_·7H_2_O.

### 2.3. Experimental Methods

#### 2.3.1. Setting Time

The Chinese standard GB175-2007 [26] is used for measuring the fresh paste’s setting time. The fresh paste is poured into the oiled mold with a frustum size whose top inner diameter is 65 mm, the bottom inner diameter is 75 mm, and the height is 40 mm. The sample is cured in the standard curing box for 30 min after water is added. Then, the initial setting time is tested following these steps: First, the mold is moved from the curing box (20 ± 2 °C and 98.3% relative humidity) and the test needle is placed in contact with the sample’s surface. After holding the needle in that position for 1–2 s, the test needle is loosened and sinks vertically and freely into the paste. Finally, the initial setting time is measured every 10 min. After the measurement of the initial setting time is finished, the testing mold is turned over immediately and put into the standard curing box to continue curing. When the measuring time is close to the final setting time (the final setting time is determined by other researchers [27]), the final setting time is tested every 5 min. The initial setting time occurs when the distance between the needle tip and test mold is 4 ± 1 mm, while the final setting time is achieved when the test needle sinks 0.5 mm into the sample.

#### 2.3.2. Slump Test

The slump flow of fresh paste is tested by an NLD-3 cement mortar fluidity tester, referring to the Chinese standard GB/T8077-2000 [28].

#### 2.3.3. CO_2_ Curing

An HC-HTX12 concrete carbonization test chamber provided by Jianyan Huace (Hangzhou) Technology Co., Ltd., Hangzhou, China, with a CO_2_ concentration of 8%, is used to create the CO_2_ curing environment. The relative humidity of the curing environment of the HC-HTX12 concrete carbonization test chamber is 50%, and the curing temperature is 20 °C.

#### 2.3.4. Mechanical Strength

The specimens are demolded after hardening and moved to the standard curing environment (20 ± 2 °C and 98.3% relative humidity) or the CO_2_ curing environment for 3 days and 28 days, respectively. After the curing is complete, the specimens are used for the determination of mechanical strengths. The mechanical strengths are tested by a YDW-100C microcomputer full-automatic universal tester. The loading speeds for the flexural and compressive strength measurements are 0.05 kN/s and 2.4 kN/s, respectively.

#### 2.3.5. Erosion Resistance

A CHFL-360 cyclic immersion corrosion tester manufactured by Wuxi Chihe Test Instrument Co., Ltd., Wuxi, China, is used for the water D-W alternations experiment. A water D-W alternation lasts 24 h. The rapid freezing and thawing tester, which is produced by Xianxian Shengshi Lutong Test Instrument Factory, Cangzhou, China, is applied to determine the F-T cycles. All specimens are immersed in water for 4 days after standard curing for 24 days. Then the specimens are moved to the freeze-thaw rubber pipes filled with water in the rapid freezing and thawing tester. The freeze-thaw temperature is between −17 ± 2 °C and 8 ± 2 °C. An F-T cycle lasts 2.5 h–4 h. An LSY-18B fully automatic concrete sulfate D-W cycle test chamber is applied to the research of sulfate D-W cycles. All the experiments are carried out according to the GB/T 50082-2009 Chinese standard [29]. The mass, flexural strength, and compressive strength are tested for the characterization of erosion resistance. The residual rates of mechanical strengths (the ratios of mechanical strengths after water D-W alternations to the strengths before water erosion) are selected to reflect the water resistance of specimens. The total repeated cycles of water D-W, F-W, and sulfate D-W are 60, 100, and 60, respectively.

#### 2.3.6. Drying Shrinkage Measurement

**Figure 1:**
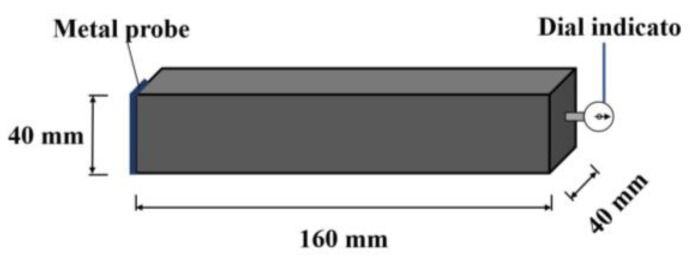
The measurement of dry shrinkage rate.

The shrinkage rod of the dial indicator is applied to measure the drying shrinkage (D-S) rate of the specimens. First, one end of the rectangular specimen is supported by the dial indicator. When the length of the specimen changes, the value of the length change is read out by the dial indicator. The measuring device is shown in Figure 1. The shrinkage (D-S) rates of the specimens are measured in an environment with 20 ± 2 °C and a relative humidity of 60%. At first, each specimen’s length (*L*_0_) is determined after the initial condensation. Then the length (*L_t_*) of each curing time for the specimen is measured. The dry shrinkage rate (*S_d_*) is calculated using Equation (1).

(1) $$S_{d}=\frac{L_{t}-L_{0}}{L_{0}}$$

#### 2.3.7. Scanning Electron Microscope Energy Spectrum

The ellipsoidal samples in the center of specimens with a maximum diameter of 2 mm and a minimum diameter of 1 mm are taken out and applied to the measurement of the Scanning Electron Microscope with Energy Dispersive Spectroscopy Detector (SEM-EDS). The samples are dried in a vacuum drying oven with a temperature of 60 °C for 3 days, then all samples are sprayed with gold and moved to the ZEISS SIGMA 300 field emission electron microscope for measurement with SEM-EDS photos. The samples are magnified 10,000 times, and the amplified photos are collected for the observation of microscopic morphology. 

#### 2.3.8. Measurement of Pore Size Distribution

The mercury intrusion method is used to analyze the pore size distribution of the MOS paste. A full-automatic mercury intrusion meter (Microtrac MRB/Mechkbair) is used to obtain the pore size distribution of a sample. The sample preparations and measurements are carried out according to Wang’s research [9].

#### 2.3.9. Measurement of Density

The specimens are ground into powder by a ball mill. After that, the powdered samples are dried in a vacuum drying oven at a temperature of 105 ± 2 °C until the mass of the sample is constant. Finally, Li’s bottle method is applied to the measurement of density. The measuring process can be found in Du’s journal [6].

In this study, three specimens are selected for the measurement of the working performance, mechanical strengths, and drying shrinkage. The average values of the three specimens are used as the measuring results. The error bar’s value is the coefficient of variation of the measured values of each group, which is the standard deviation divided by the average.

## 3. Results and Discussions

In Figure 1 to this article, the column graphs in Figure 2, Figure 3, Figure 4, Figure 5, Figure 6, Figure 7, Figure 8, Figure 9, Figure 10, Figure 11 and Figure 12 represent the measuring values, and the line charts are the varying rates of the experimental parameters.

### 3.1. Setting Time

The setting times of fresh MOS paste are illustrated in Figure 1. The setting times increase as the dosage of citric acid increased. This is because a protective film is formed by the citric acid, which leads to an inhibited hydration reaction in the paste and increases the setting time [5]. The increasing rates of the initial setting time caused by citric acid range from 0% to 124.8%, while the final setting time caused by citric acid is from 0% to 113.4%. The increasing rate of the initial setting time caused by citric acid is higher than that of the final setting time. This is because the addition of citric acid can decrease the hydration heat of MOS during the derivative period of the hydration process [30]. Consequently, the MOS shows a higher increasing rate for the initial setting time than that of the final setting time. The error bar values are less than 10% of the total values, which shows the accuracy of the determining values.

**Figure 2:**
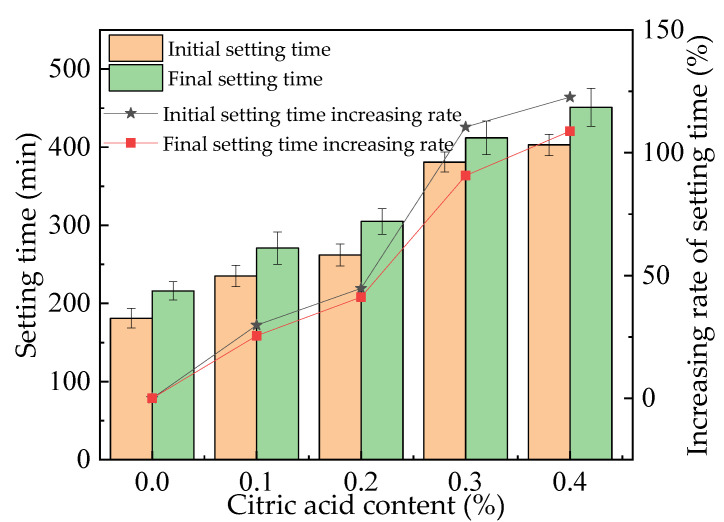
The setting time of the fresh MOS paste.

### 3.2. Slump Flow Test

**Figure 3:**
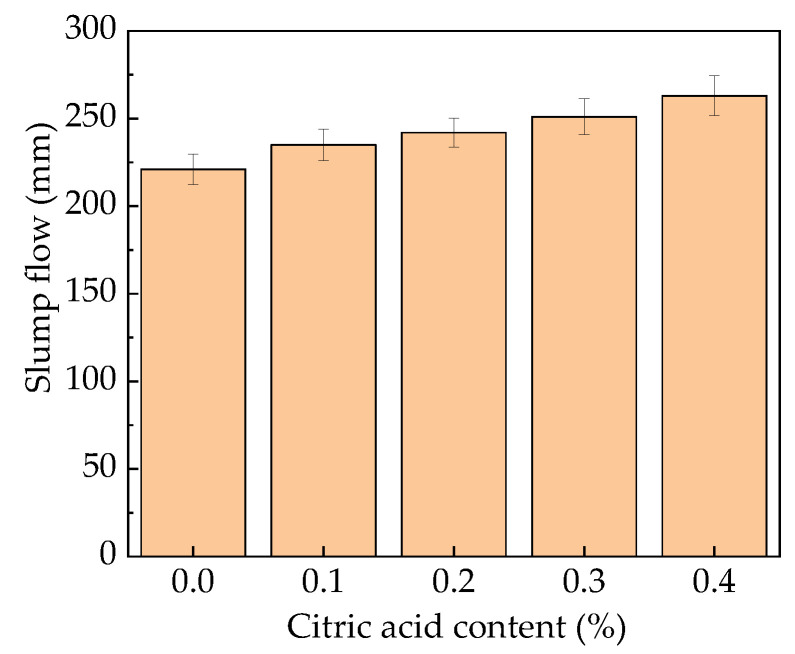
The slump flow of the fresh MOS paste.

The slump flow of the fresh MOS paste is shown in Figure 3. The slump flow increases with the increasing dosages of citric acid. When the dosage of citric acid varies from 0% to 0.4%, the slump flow increases from 221 mm to 263 mm. This is attributed to the fact that the citric acid can promote the dispersion of MOS particles, thus increasing the slump flow of the fresh MOS paste [11].

### 3.3. Mechanical Properties

**Figure 4:**
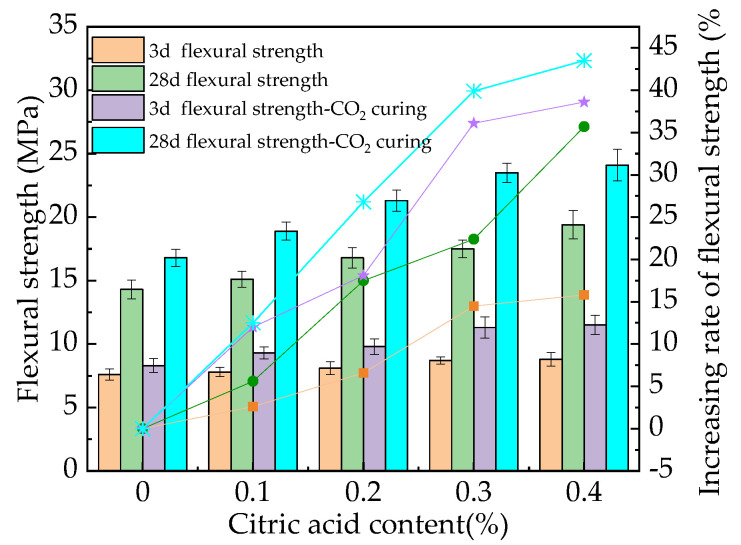
The flexural strength of the MOS paste.

Figure 4 shows the flexural strength and the corresponding increasing rates of the MOS paste. The flexural strength increases as the citric acid dosage increases. This is attributed to the fact that citric acid can reduce the hydration heat of MOS, thus decreasing the cracks in the inner MOS [31]. Moreover, citric acid leads to an increase in the 5Mg(OH)_2_·MgSO_4_·7H_2_O phase, which is more compact than the normal products (3Mg(OH)_2_·MgSO_4_·8H_2_O) [32]. Consequently, the flexural strength of MOS is improved by the addition of citric acid. Finally, the CO_2_ curing demonstrates a positive effect on the flexural strength of the MOS paste due to the formation of magnesium carbonate by the reaction of the magnesium oxide and CO_2_ [22]. Figure 4 shows that the maximum increasing rate of flexural strength by citric acid ranges from 16.8% to 44.2%. Meanwhile, the CO_2_ curing on MOS increases the flexural strength by 12.4% to 37.1%. This is attributed to the fact that the CO_2_ curing can increase the content of magnesium carbonate, thus improving the compactness and mechanical strength of the MOS paste [19].

**Figure 5:**
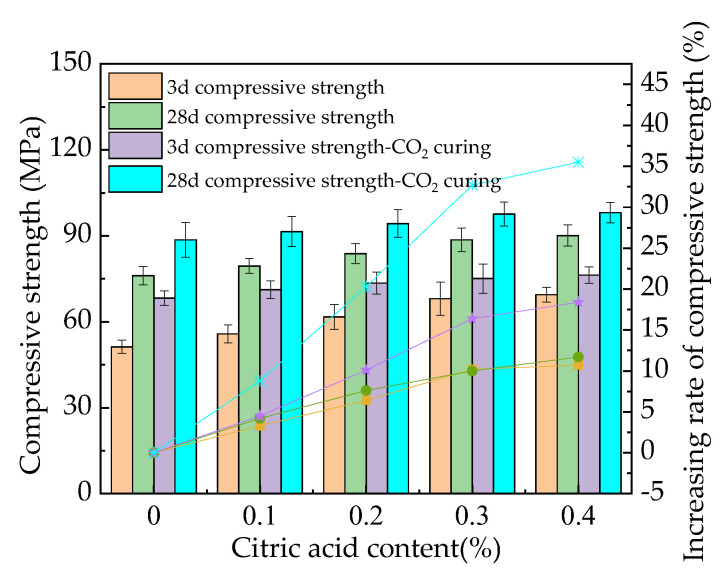
The compressive strength of the MOS paste.

Figure 5 presents the compressive strength and the corresponding increasing rates. The trend of compressive strength is the same as that of flexural strength. The compressive strength and the corresponding varying rates are improved by the dosages of citric acid and the CO_2_ curing. Figure 5 shows that the maximum changing rate of compressive strength caused by citric acid ranges from 11.3% to 36.3%. Meanwhile, the CO_2_ curing on MOS increases the compressive strength by 8.7% to 35.2%. These are attributed to the same reasons as the flexural strength. Comparing the compressive strength with the flexural strength, the increasing rates of flexural strength by citric acid content and CO_2_ curing are higher than those of the compressive strength. The error values of the mechanical strengths are all lower than 8.4% of the total values, indicating the precision of the experimental results. Finally, as shown in Figure 4 and Figure 5, the flexural and compressive strengths of MOS paste cured for 28 days are higher than those cured for 3 days due to the improved hydration caused by the increased curing age. Compared to earlier research [33], the MOS paste cured in the standard curing environment with citric acid showed better mechanical strength.

### 3.4. Drying Shrinkage Rate

**Figure 6:**
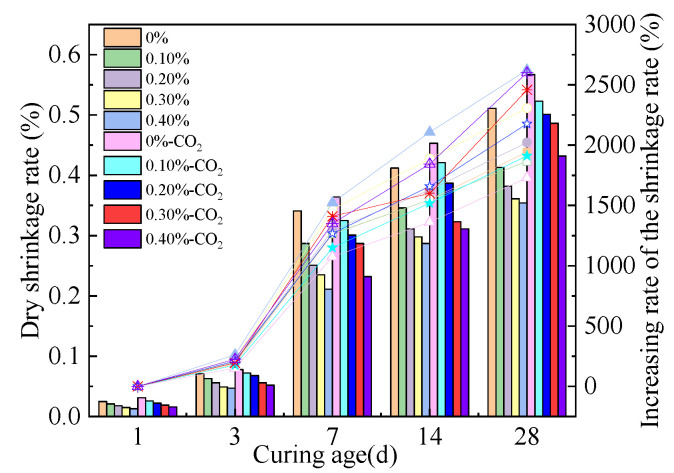
The D-S rate of the MOS paste.

The D-S rate of the MOS paste is shown in Figure 6. The increasing curing age and the citric acid lead to an increased D-S rate. This is because the citric acid can delay the setting time of the MOS paste [12]. Therefore, the water loss rate of hydration is reduced by the increasing dosages of citric acid. Moreover, the D-S rate of the MOS paste is increased by the CO_2_ curing due to the fact that the humidity of the carbonization environment is 50%, which is lower than the humidity of the standard environment (98.3% relative humidity), which leads to an increase in the D-S rate of the MOS paste [34].

### 3.5. Water Resistance

**Figure 7:**
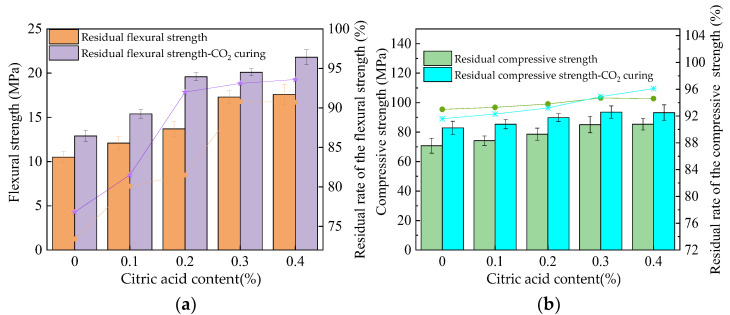
The residual strength of the MOS paste after water immersion. (**a**) Flexural strength. (**b**) Compressive strength.

The residual strength of the MOS paste after immersion in water for 4 days is shown in Figure 7. The mechanical strength can be decreased by the erosion caused by the water. This is because the hydration products of the MOS phase are relatively loose and easy to penetrate in the water, so they will encounter water corrosion [35]. As shown in Figure 7, the residual compressive and flexural strengths increase with the increasing dosages of citric acid. The CO_2_ curing process can effectively improve the residual mechanical strengths of MOS paste. Due to the fact that the MOS has poor water resistance, the CO_2_ curing can effectively improve the compactness by increasing the content of magnesium carbonate. The residual rates of the residual flexural and compressive strengths are increased from around 73.1–76.3% and 92.8–93.6% to around 90.8–92.1% and 93.8–96.1%, respectively, by adding citric acid. Results show that the flexural strength of the MOS paste attenuates more than the compressive strength. The CO_2_ curing can improve the residual rate of flexural strength from 73.1–90.3% to 77.2–92.1%. CO_2_ curing can improve the residual rate of the compressive strength from 91.3–94.2% to 94.1–95.8%. Compared with other studies, the water resistance of CO_2_-cured MOS paste with citric acid is better than that of just MOS paste with citric acid.

**Figure 8:**
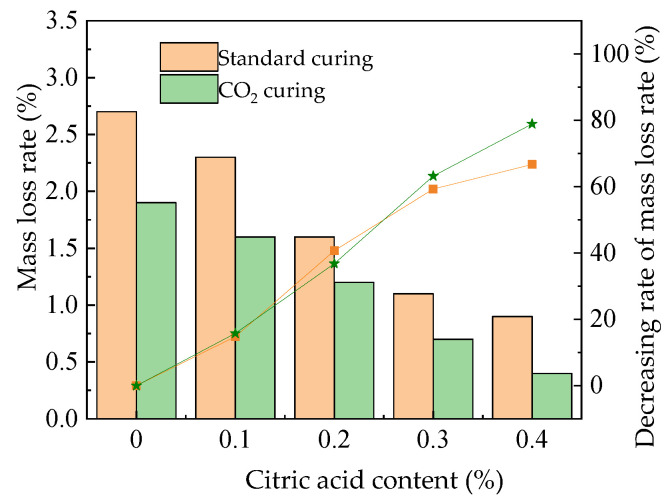
The mass loss rate after water immersion.

The mass loss rate of the MOS paste is illustrated in Figure 8. The mass loss rate of the MOS paste presents a decreasing trend with the increasing dosages of citric acid. The CO_2_ curing has a decreasing effect on mass loss. This is attributed to the fact that the resistance to water erosion is improved by the addition of citric acid and CO_2_ curing, as analyzed in the description in Section 3.3. The mass of surface spalling decreases during the water effect, eventually leading to a decrease in the mass loss.

### 3.6. Freeze-Thaw Resistance

**Figure 9:**
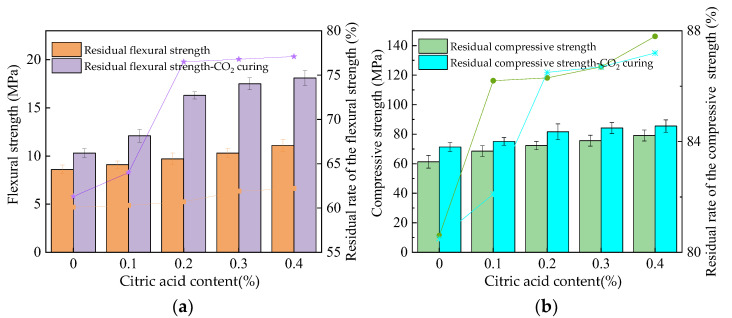
The residual strength of the MOS paste after F-T cycles. (**a**) Flexural strength. (**b**) Compressive strength.

Figure 9 depicts the residual strengths of the MOS paste after 100 F-T cycles. The flexural and compressive strengths of specimens after 100 F-T cycles are 58.3–60.1%, compared to 78.6–87.3% of the specimens before the F-T cycles. Meanwhile, when the specimens are cured in a CO_2_ curing environment, the flexural and compressive strengths of the specimens after 100 F-T cycles are 59.1–76.3%, compared to 81.6–87.5% of the specimens before the F-T cycles. This is because the erosion of F-T cycles can accelerate crack propagation in the inner specimens, resulting in mechanical strength attenuation [36]. It can be observed in Figure 9 that the addition of citric acid content can improve the residual strengths of MOS. This is ascribed to the fact that the compactness of the hydration products can be increased by citric acid, resulting in an improvement in mechanical strengths. The content of MgCO_3_ can be increased through the reaction of CO_2_ and Mg(OH)_2_ when CO_2_ curing is performed. MgCO_3_ absorbs on the surface of MOS hydration products, which improves their compactness and corresponding mechanical strengths.

**Figure 10:**
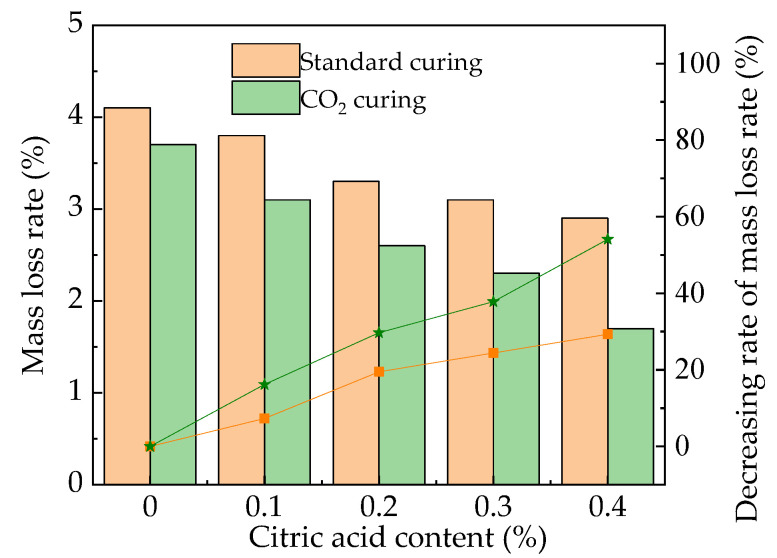
The mass loss rate of the MOS paste after F-T cycles.

The mass loss rate of the MOS paste after 100 F-T cycles is illustrated in Figure 10. It can be observed in Figure 10 that the mass loss rates show a decreasing trend with the increase in citric acid content. When the addition of citric acid content increases from 0% to 0.2%, the decreasing rates of standard curing MOS paste and CO_2_ curing MOS paste are 18.8% and 32.3%, respectively. When the dosage of citric acid increases from 0.2% to 0.4%, the decreasing rates are 30.1% and 56.3%, respectively. As observed in Figure 10, the CO_2_ curing can further demonstrate the decreasing effect on the mass loss rate of the MOS paste after water F-T cycles. This is attributed to the fact that the addition of citric acid can improve the compactness of the hydration products of MOS paste after longer curing (more than 28 days) [5]. Therefore, the peeling of the specimens during freezing and thawing is reduced.

### 3.7. Sulfate Erosion

**Figure 11:**
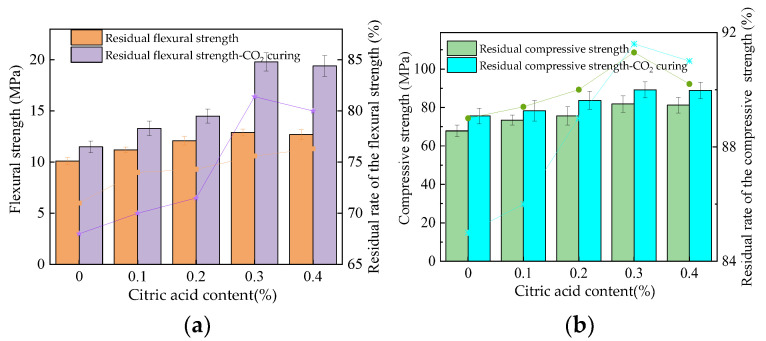
The residual strength of the MOS paste after 60 sulfate dry-wet alternations. (**a**) Flexural strength. (**b**) Compressive strength.

The residual strengths of the MOS paste after 60 sulfate D-W cycles are shown in Figure 11. The mechanical strengths of specimens after 60 sulfate D-W cycles are 62.3–64.1%, compared to 82.6–88.1% of the specimens before sulfate D-W cycles. Additionally, the flexural and compressive strengths of CO_2_ cured specimens decreased to 63.4–67.3% and 82.6–89.1%, respectively, by the sulfate D-W cycles. The crystallization force of sulfate crystals is produced during the dry-wet cycle [37], leading to expansion damage caused by the sulfate attack and decreasing the mechanical strengths of the MOS. As illustrated in Figure 11, the addition of citric acid and CO_2_ curing can effectively improve the residual mechanical strength of MOS paste. This is because the citric acid can reduce the early hydration heat and delay the formation of internal cracks [38]. The MgCO_3_ formed by CO_2_ can prevent inner specimen cracking, which demonstrates positive effects on the mechanical strengths during sulfate D-W cycles.

**Figure 12:**
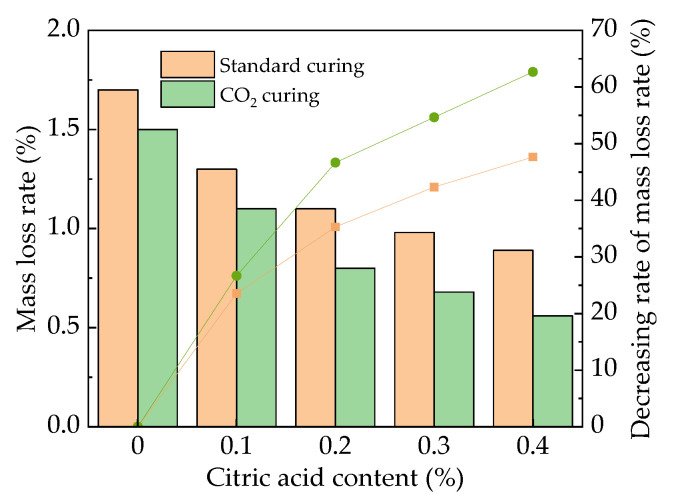
The mass loss rate of the MOS paste after 60 sulfate dry-wet alternations.

The mass loss rate of the MOS paste is shown in Figure 12. The mass loss rate decreases with the addition of citric acid and CO_2_ curing. The sulfate erosion effect leads to the surface peeling and decreases the mass of the specimens. The addition of citric acid can also reduce the inner cracks of specimens, and the CO_2_ curing can improve the compactness, thus decreasing the mass loss rates. It can be summarized from all the results that the effect of sulfate corrosion on the samples is small.

### 3.8. Scanning Electron Microscope

**Figure 13:**
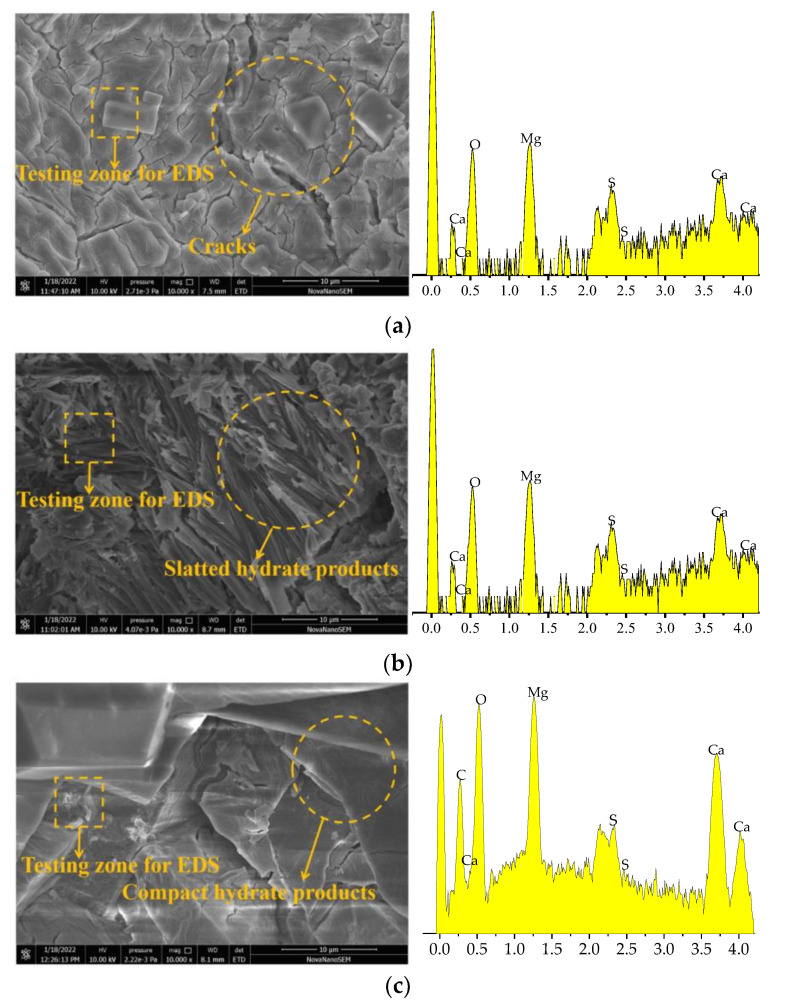
SEM-EDS of specimens. (**a**) 0% citric acid. (**b**) 0.2% citric acid. (**c**) 0.2% citric acid-CO_2_ cured.

SEM-EDS of specimens with 0% and 0.2% citric acid are shown in Figure 13a,b. Figure 13c shows the SEM-EDS of the CO_2_ cured-specimen with 0.2% citric acid. All the specimens are standard-cured for 28 days. As observed in Figure 13b, the slatted hydrate products are found. Several cracks are discovered in Figure 13a. Comparing Figure 13a with Figure 13b, the inner cracks of MOS are decreased by adding citric acid. Comparing Figure 13b with Figure 13c, the hydration products in Figure 13c are more compact. CO_2_ curing can effectively improve the compactness of hydration products. As reported in prior studies, materials with more dense hydration products show higher mechanical strengths [39]. In combination with the above research results, it can be concluded that increasing the dosages of citric acid and CO_2_ curing can effectively increase the mechanical strength and erosion resistance of the MOS paste. Table 2 shows the element distribution obtained by EDS results. The amount of Mg element is increased by adding citric acid, and CO_2_ curing leads to improved amounts of C element.

### 3.9. Porosity Analysis

**Figure 14:**
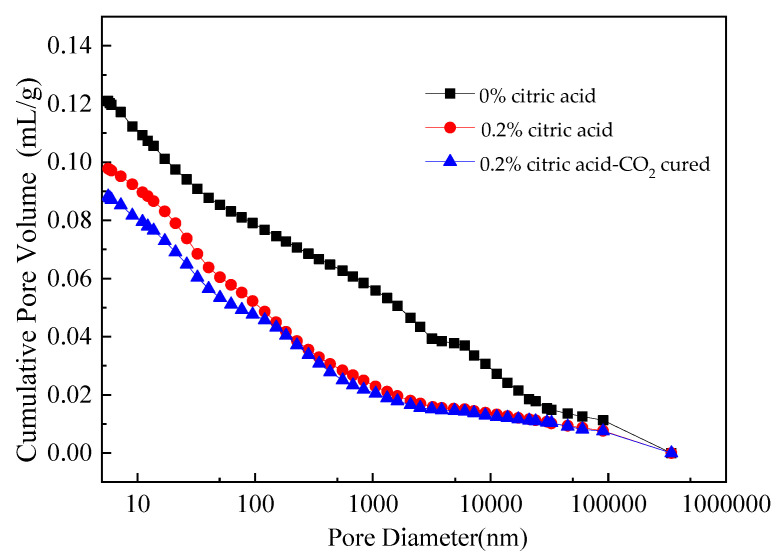
Relationship between pore size and mercury intake.

The relationship between the pore diameter and the cumulative pore volume of MOS paste without citric acid, MOS paste with 0.2% citric acid, and samples with 0.2% citric acid and cured in a CO_2_ environment is shown in Figure 14. It can be observed in Figure 14 that the main pore diameters of the MOS paste with 0% citric acid, 0.2% citric acid, and 0.2% citric acid-CO_2_ cured are 4.12–8.56 × 10^5^ nm, 3.26–2.56 × 10^5^ nm, and 2.58–1.98 × 10^5^ nm, respectively. As reported in prior studies, cement-based materials with a smaller pore diameter and cumulative pore volume have higher mechanical strengths and durability [40]. Therefore, the result of the porosity analysis further confirms that the addition of citric acid and CO_2_ curing can improve the mechanical strengths as well as the resistance to water immersion and F-T cycles. Table 3 shows the density of MOS paste. As shown in Table 3, the addition of citric acid and CO_2_ curing can increase the density of MOS paste, thus increasing its mechanical strength and durability.

## 4. Conclusions

In this study, we investigated the influence of citric acid on the working and mechanical properties of CO_2_-cured magnesium oxysulfate (MOS) paste. Overall, the following conclusions can be summarized:

The results show that as the dosage of citric acid increases from 0% to 0.4%, the initial setting time of MOS paste increases by 0% to 124.8%, and the final setting time increases by 0% to 113.4%. The rate of slump flow also increases, from 221 mm to 263 mm.

Additionally, the flexural and compressive strengths of MOS paste increase by 16.8% to 44.2% and 11.3% to 36.3%, respectively. CO_2_ curing can further increase these strengths by 12.4% to 37.1% and 8.7% to 35.2%, respectively. The drying shrinkage rate of MOS paste decreases with the addition of citric acid but increases with CO_2_ curing.

When exposed to water erosion, the residual strength of the MOS paste improves with increased citric acid and CO_2_ curing. After 100 freeze-thaw cycles, the residual strength also improves with the addition of citric acid and CO_2_ curing, and the erosion effect is less severe with sulfate D-W alternations compared to water D-W alternations and F-W cycles. The best erosion resistance is seen with 0.3% citric acid, and CO_2_ curing and citric acid help to compact the hydration products.

This research provides a new kind of cement-based material (CO_2_-cured MOS) for the construction of buildings. Performance enhancement and CO_2_ emission reduction methods can be applied in the manufacturing process of cement-based materials.

## Figures and Tables

**Table 1:** The mixing proportions of MOS paste (g).

Speciemens	MgO	MgSO_4_·7H_2_O	Water	Citric Acid
MOS-0	422	178	143.5	0
MOS-0.1	422	178	143.5	0.6
MOS-0.2	422	178	143.5	1.2
MOS-0.3	422	178	143.5	1.8
MOS-0.4	422	178	143.5	2.4

**Table 2:** The element distribution obtained by EDS (%).

Elements	Mg	O	S	C
0% citric acid	39.09	9.82	50.61	0.48
0.2% citric acid	57.1	4.15	38.39	0.36
0.2% citric acid-CO_2_ cured	57.97	14.49	23.19	4.35

**Table 3:** The density of MOS (g/cm^3^).

With 0% Citric Acid	With 0.2% Citric Acid	With 0.2% Citric Acid-CO_2_ Cured
3.21	3.34	3.37

## Data Availability

The data used to support the findings of this study are available from the corresponding author upon request.
